# The influence of study population and definition of improvement on the smallest detectable change and the minimal important change of the neck disability index

**DOI:** 10.1186/1477-7525-12-53

**Published:** 2014-04-15

**Authors:** Wouter Schuller, Raymond WJG Ostelo, Richard Janssen, Henrica CW de Vet

**Affiliations:** 1 VU Medical Center, EMGO+ Institute for Health and Care Research, van der Boechorststraat 7, 1081 BT Amsterdam, the Netherlands; 2 Private practise for OrthoManual Medicine, Stationsstraat 60, 1506 DH Zaandam, the Netherlands; 3 Private practise for OrthoManual Medicine, Klein Haasdal 4, 6333 AK Schimmert, the Netherlands

**Keywords:** Smallest detectable change, Minimal important change, Neck disability index, Global perceived effect, Reliability, Measurement properties

## Abstract

**Background:**

Reported values of the minimal important change (MIC) and the smallest detectable change (SDC) for the neck disability index (NDI) differ strongly, raising questions about the generalizability of these parameters. The SDC and the MIC are possibly influenced by the study design or by the study population. We studied the influence of the type of anchor, the definition of improvement and population characteristics on the SDC and the MIC of the NDI.

**Methods:**

A cohort study including 101 patients with non-specific, chronic neck pain. SDC and MIC were calculated using two types of external anchors. For each anchor we applied two different definitions to dichotomize the population into improved and unimproved patients. The influence of patient characteristics was assessed in relevant subgroups: patients with or without radiating pain and patients with different baseline scores.

**Results:**

The influence of different anchors and different definitions of improvement on estimates of the SDC and the MIC was only minimal. The SDC and the MIC were similar for subgroups of patients with or without radiation, but differed strongly for subgroups of patients with higher or lower baseline scores.

**Conclusions:**

Our study shows that estimates of the SDC and the MIC of the NDI can be influenced by population characteristics. It is concluded that we cannot adopt a single change score to define relevant change by combining the result of previous studies.

## Introduction

The Neck Disability Index (NDI) was published by Vernon in 1991 as a patient reported outcome measure of disability in patients with neck pain [1]. It has been reported to be the most commonly used self-report instrument for evaluating functional status in neck pain clinical research [2,3]. A review published in 2008 stated that the NDI had been used in approximately 300 publications, and translated into 22 languages [4]. Many studies have addressed measurement properties of the NDI, and two systematic reviews of these studies were recently published. In a review by MacDermid et al. three comprehensive review articles and 41 studies that addressed at least one psychometric property were identified [2]. The authors concluded that the NDI is reliable, valid and responsive in various patient populations, including patients with acute and chronic conditions, as well as those suffering from neck pain associated from musculoskeletal dysfunction, whiplash-associated disorders, and cervical radiculopathy. The authors stated that the work on smallest detectable change (SDC) and minimal important change (MIC) is sparse and inconsistent but followed Vernon in suggesting an accepted MIC of 5 (10%) [2]. In a more recently published systematic review by Schellingerhout et al. [5] the COSMIN checklist [5] was used to assess the quality of the collected studies. These authors concluded that the NDI scored positive on internal consistency, content validity, structural validity, hypothesis testing and responsiveness, but showed low reliability. According to Schellingerhout et al. a value for the MIC cannot be provided yet, as the estimates for the MIC are too diverse [6], and more studies are needed that determine the SDC and the MIC for the NDI in different subgroups of patients.

The SDC and the MIC are considered important parameters to enable a proper interpretation of change scores [7]. Studies that reported the SDC and the MIC for the NDI are presented in Table 1[8-14]. In these studies different patient populations were recruited, and different follow-up periods and different definitions of improvement on the anchor were used, reporting MIC values ranging from 3.5 to 9.5, and SDC values ranging from 3.0 to 17.9. This strong variation in reported MIC and SDC raises questions about the generalizability of these parameters. Can we adopt a generalized value for the MIC and the SDC by combining the results of previous studies, or should we estimate these parameters for different populations separately? To get a better insight in the possible causes of this variation one could study the influence of different study designs or different study populations on the SDC and MIC in a single study. We assessed the influence of the type of anchor, the definition of improvement and of population characteristics on the SDC and the MIC of the NDI in a single population of patients with general, non-specific, chronic neck pain.

**Table 1:** **Previous studies presenting estimates of SDC and MIC**^**a**^

**Study**	**NP/CR**^**a**^	**Population**^**a**^	**N**	**NDI (T**_**0**_**)**	**GPE**	**Stable**	**SDC**	**MIC (sens/spec)**
Cleland [9]^b^	NP	acute + chronic	137	32.2-35.7	15 pt	-3 to +3	11.6*	9.5 (0.83/0.72)
Young [14]^b^	CR	average 4 weeks	165	24-25	13/15 pt	-1 to +1	17.9*	8,5 (0.62/0.79)
Young [13]	NP	unclear	91	15.8-18.0	15 pt	-2 to +2	12.1*	7.5 (n.r.)
Cleland [9]^b^	NP	acute + chronic	137	32.2-35.7	15 pt	-2 to +2	8.1	7.0
Cleland [8]	NP + CR	average 2 weeks	38	21.9-20.7	15 pt	-3 to +3	12.0	7.0 (0.52/0.59)
Young [14]^b^	CR	average 4 weeks	165	24-25	13/15 pt	-2 to +2	15.9*	6.5 (0.57/0.67)
Jorritsma [15]	NP	chronic	76	21	7 pt	-1 to +1	8.4	3.5
Pool [10]	NP	acute + chronic	183	14.5	6 pt	2 to 4	10.5	3.5 (0.90/0.70)
Vos [12]^c^	NP	acute	187	13.0-16.0	7 pt	3 to 5	7.62	n.r.^a^
Trouli [11]^d^	NP	acute + chronic	65	n.r.	15 pt	-3 to +3	3.03	n.r.^a^

Notes:

^a^NP = neck pain, CR = cervical radiculopathy, n.r. = not reported, chronic neck pain > 6 weeks;

^b^Cleland 2008 and Young 2010 reported two different analyses, these analyses are represented in the table as separate studies;

^c^Vos et. al. reported a different SDC of 1.66 in a published paper [16]. The correct SDC reported in his dissertation [12] is presented;

^d^Trouli et. al. reported a SDC of 1.78 but this value does not agree with the Bland & Altman plot presented. We present a SDC estimated from the Bland & Altman plot;

*reported SDC(90) was changed to SDC(95) (SDC(95) = 1.96 × SDC(90)/ 1.65);

## Methods

### Design

From March to October 2009 patients with general, non-specific, chronic neck pain were recruited in four practices for OrthoManual Medicine in the Netherlands. Inclusion criteria were chronic non-specific neck pain, defined as neck pain existing at least 3 months, aged 18 years or older, and having no contraindications for manipulative treatment. Duration of complaints, age, radiation into the arm(s), and the presence of concomitant headache were recorded. After signing an informed consent form patients filled in the NDI as a baseline measure (T_0_). Patients were treated by one of six OrthoManual physicians. Usually the treatment would be completed within three months, but some patients returned for treatment after this period due to persistent or recurring complaints. After a follow-up period of six months patients were asked by email to fill in the NDI questionnaire again together with questions about the global perceived effect relating to pain and to function (T_1_).

### The neck disability index

The NDI contains ten items. Seven items are related to activities of daily living, two are related to pain, and one item is related to concentration. Each item is scored on a 0-5 scale, adding up to an overall score ranging from 0 to 50, with higher scores corresponding to more severe disability. A series of studies has disputed the unidimensionality of the NDI, and various changes have been suggested to improve the unidimensionality [17-21], but this has not led to the widespread application of the suggested changes. In our study we have therefore used the NDI as a unidimensional construct.

### Type of anchor, definition of improvement and choice of clinical subgroups

Global perceived effect (GPE) was used as the external criterion and was phrased to question either change of pain or change of function, with the following possible scores: completely recovered (1), much improved (2), slightly improved (3), no change (4), slightly worse (5), or much worse (6). To assess the influence of the type of anchor we used two external anchors: one anchor referring to change of *pain* (GPE_pain_) and one anchor referring to change of *function* (GPE_function_). In addition we used two different definitions of improvement. Firstly we defined both completely recovered and much improved (GPE_1-2_) as improved, whilst defining slightly worse, no change and slightly better (GPE_3-5_) as unchanged. Patients reporting much worse (GPE_6_) were excluded from the analysis. Secondly we defined patients reporting completely recovered, much improved, and slightly improved (GPE_1-3_) as improved, only categorizing patients reporting no change (GPE _4_) as unchanged. Patients reporting slightly worse or much worse (GPE _5-6_) were excluded from the analysis. Using these two types of anchor and these two definitions of improvement enabled us to compare four different combinations of the type of anchor and the definition of improvement:

a. GPE_-pain_, unchanged = GPE_4_

b. GPE_-pain_, unchanged = GPE_3-5_

c. GPE_-function_, unchanged = GPE_4_

d. GPE_-function_, unchanged = GPE_3-5_

For each combination we have presented means and standard deviations of the NDI scores at T_0_ and at T_1_, and of the changes in score between T_0_ and T_1_. For each combination we calculated the SDC and the MIC.

To assess the influence of different clinical characteristics we considered the following subgroups of patients:

a. Patients with or without radiation. Patients with neck pain frequently have symptoms that radiate into the arm(s), while the NDI is designed for measuring neck specific disability. The NDI is also used to measure change in groups of patients with cervical radiculopathy, who could have arm symptoms without neck pain [22].

b. Patients with baseline scores above or below the median NDI score of 24 points (48%). Different baseline scores have been shown to influence measurement properties in other instruments [23]. In order to maximise the group size and thus optimize the statistical power we chose to use the median NDI score to divide our population into two groups with higher or lower baseline scores.

For each subgroup we calculated the SDC and the MIC. A GPE score of 3-5 was used to define stable patients in order to increase the number of unchanged patients.

### Analyses

– SDC was based on the standard error of measurement (SEM) which was derived from the variance component in the formula for the intraclass correlation coefficient, ICC_agreement_[24]. It was calculated on the group of patients who were considered to be unchanged by 1.96 × √2 × SEM_agreement_[25-27] (SDC 95)

– MIC was calculated using a receiver operating characteristics (ROC) curve to establish the optimal cut-off point. The optimal cut-off point was defined as the point on the ROC curve with the highest sum of specificity and sensitivity, minimizing the overall misclassification [28]. We report the MIC and the sensitivity and specificity at the cut-off point.

## Results

### Overall results

A total of 101 patients were recruited and gave informed consent, of whom 99 patients completed the follow up measurement. Patients’ characteristics are presented in Table 2. The mean age at inclusion was 42 years (SD±12 years). The average duration of complaints was 77 months (range 2-480, median 24 months). More than half of the patients (N = 54) reported that the pain radiated into the arm(s) and 78% of patients reported concomitant headache. For both external anchors, and for each definition of improvement, the mean scores and the standard deviations at T_0_, at T_1_, and of the change scores are presented in Table 3. A clear trend can be seen of an increasing change score in accordance with both GPE scores. Spearman correlation was used because the GPE scores were considered to be not normally distributed on a histogram. Correlation coefficients between the NDI change score and the GPE for pain and the GPE for function were 0.60 and 0.58 respectively.

**Table 2:** Patient characteristics at inclusion (N = 101)

Mean age at inclusion (range)	42 (19-71)
Mean duration of complaints in months (range)	77 (2-480)
Mean NDI score at baseline (SD)	24.4 (6.2)
Radiating pain	54%
Headache	78%

**Table 3:** NDI scores at baseline and after 6 months for different levels of the GPE (N = 99)

**GPE pain**	**NDI:T**_**0**_**, mean (sd)**	**NDI:T**_**1**_**, mean (sd)**	**NDI:T**_**1**_**-T**_**0**_**, mean (sd)**
1 Completely recovered (N = 14)	22.1 (3.1)	11.3 (1.9)	-10.9 (3.6)
2 Much improved (N = 41)	24.0 (6.8)	16.5 (3.3)	-7.5 (6.0)
3 Slightly improved (N = 17)	25.1 (5.4)	22.0 (4.8)	-3.1 (4.0)
4 Unchanged (N = 23)	25.6 (7.1)	24.6 (9.1)	-1.0 (5.9)
5 Slightly worse (N = 2)	28.0 (7.1)	32.0 (1.5)	4.0 (8.5)
6 Much worse (N = 2)	27.0 (0.0)	27.0 (9.9)	0.0 (9.9)
GPE 1-3 (N = 72)	23.9 (6.0)	16.8 (5.0)	-7.1 (5.8)
GPE 4 (N = 23)	25.6 (7.1)	24.6 (9.1)	-1.0 (5.9)
GPE 5-6 (N = 4)	27.5 (4.1)	29.5 (6.5)	2.0 (7.8)
GPE 1-2 (N = 55)	23.5 (6.1)	15.6 (3.8)	-8.4 (5.7)
GPE 3-5 (N = 42)	25.5 (6.3)	23.9 (7.6)	-1.6 (5.4)
GPE 6 (N = 2)	27.0 (0.0)	27.0 (9.9)	0.0 (9.9)
**GPE function**	**NDI:T**_**0**_**, mean (sd)**	**NDI:T**_**1**_**, mean (sd)**	**NDI:T**_**1**_**-T**_**0**_**, mean (sd)**
1. Completely recovered (N = 19)	22.7 (6.5)	12.3 (2.6)	-10.5 (6.9)
2. Much improved (N = 34)	24.3 (6.2)	16.9 (3.4)	-7.5 (4.7)
3. Slightly improved (N = 17)	25.0 (5.5)	22.1 (4.5)	-3.0 (3.4)
4. Unchanged (N = 26)	25.0 (6.8)	23.5 (9.1)	-1.6 (5.9)
5. Slightly worse (N = 2)	25.0 (2.8)	33.5 (0.7)	8.5 (2.1)
6. Much worse (N = 1)	32.0	32.0	
GPE 1-3 (N = 70)	24.1 (6.0)	16.9 (4.9)	-7.2 (5.7)
GPE 4 (N = 26)	25.0 (6.8)	23.5 (9.1)	-1.6 (5.9)
GPE 5-6 (N = 3)	27.3 (4.5)	33.0 (1.0)	5.7 (5.1)
GPE 1-2 (N = 53)	23.8 (6.3)	15.2 (3.8)	-8.6 (5.7)
GPE 3-5 (N = 45)	25.0 (6.1)	23.4 (7.8)	-1.6 (5.4)
GPE 6 (N = 1)	32.0	32.0	0.0

### Influence of the type of anchor and of the definition of improvement

The values of the SDC and the MIC are presented in Table 4, together with the sensitivity and specificity at the cut-off point of the ROC curve. Comparing the four different combinations of the type of anchor and the definition of improvement reveals only minimal differences. The SDC ranged from 10.6 to 11.4. The MIC is 2.5, independent of the anchor used (questioning pain or function), and independent of the way in which unchanged patients were defined (GPE_4_ or GPE_3-5_).

**Table 4:** SDC and MIC using two different anchors and two different ways to dichotomize GPE scores (N = 99)

		**SDC**	**MIC**	**Sensitivity**	**Specificity**
Pain	Unchanged = GPE 4 (N = 23)	11.5	2.5	0.739	0.806
	Unchanged = GPE 3-5 (N = 42)	11.0	2.5	0.690	0.927
Function	Unchanged = GPE 4 (N = 26)	11.8	2.5	0.731	0.829
	Unchanged = GPE 3-5 (N = 45)	11.0	2.5	0.644	0.925

### Influence of clinical characteristics

Analyses for subgroups of patients are presented in Table 5. We chose to carry out these analyses using the external anchor referring to change of pain only, because we found minimal difference in our estimates between the differently phrased external anchors, and the GPE questioning improvement of pain is frequently used in other studies. Patients with or without radiation had a similar SDC and the MIC (11.0 and 2.5 respectively). The SDC and the MIC were different for patients with baseline NDI scores above or below 24. With a baseline score < 24 the SDC was 5.1 and the MIC 2.5. With a baseline score ≥ 24 the SDC was 10.3 and the MIC 4.0.

**Table 5:** Clinical subgroup analysis of SDC and MIC for the NDI. GPE on pain (3-5 = unchanged)

	**SDC**	**MIC**	**Sensitivity**	**Specificity**
No radiation (N = 47)	11.0	2.5	0.750	0.889
Radiation (N = 54)	11.0	2.5	0.654	0.963
Baseline < 24 (N = 49)	6.8	2.5	0.882	0.871
Baseline ≥24 (N = 52)	13.0	4.0	0.600	1.000

## Discussion

### Summary of findings

Using different types of anchors or applying different definitions of improvement had a minimal influence on our estimates of the SDC and the MIC. Estimates of the SDC and the MIC were similar for patients with or without radiation, but differed for patients with different baseline scores, with a cut-off NDI score of 24 points (48%). Patients with a baseline score above 24 points had a higher MIC than patients with a lower baseline score. The SDC is similar in almost all analyses but again varies strongly in patients with higher or lower baseline scores. It can be concluded that the different estimates of SDC and MIC in our study are predominantly explained by patient characteristics.

### Comparison with previous studies

Differences in population characteristics influence estimation of the SDC and the MIC of the NDI. Could different patient characteristics alone explain the different estimates reported in previous studies? The results of these studies are presented in Table 1, ranked according to the estimated MIC. Clearly the study of Cleland et al. [9] with the highest baseline NDI does have the highest MIC (9.5), but in this study the MIC was reduced to 7.0 with a narrower definition of unchanged patients. Studies recruiting patients with cervical radiculopathy regardless of the coexistence of neck pain have the highest estimates of the SDC, but patients with cervical radiculopathy do not necessarily have neck pain as well [22]. The perceived effect in these patients could be related to arm symptoms, while the NDI specifically measures neck symptoms. It is likely that this would reduce the correlation between the NDI score and the GPE, thus increasing the variance of the change scores in the group of stable patients. This would lead to higher estimates of the SDC and decrease the sensitivity and the specificity of the MIC. In our study we did not observe any difference in SDC between the subgroup of patients with or without radiating pain. This could be explained by the fact that we recruited a population of patients with neck pain, and we did not specifically screen for radiculopathy. Judged by the high SDC’s and the low sensitivity and specificity of the MIC in populations with a primary complaint of cervical radiculopathy it does seem that the NDI is less useful in these populations.

Due to the different populations recruited and the different methods used it remains very difficult to compare the estimates from previous studies. In our view different patient populations indeed seem to lead to different estimates of the SDC and of the MIC, but it is unclear whether patient characteristics are the only source explaining these differences. The dual factor structure of the NDI could possibly explain part of these findings. This might be improved by changing the NDI to a shorter version with an improved factor structure, as suggested by several authors [17-21], but it could also be that other instruments for measuring neck related disability will eventually prove more useful. It is clear that the SDC is much higher than the MIC in most studies, ranging even to 17.9 in a population with cervical radiculopathy. Given this high SDC one needs a change score much higher than the MIC to reliably label a patient as improved. This raises questions about the usefulness of the NDI to assess change in individual patients [24].

Although we did not study the influence of the follow-up period it is interesting to see that the MIC in our study is rather low compared to most other studies, despite our long follow-up period of six months. This could possibly be explained by the strict recruitment of patients with chronic neck pain. Selecting a population with chronic neck pain could have excluded patients with a favourable short term outcome whilst including patients with more stable and unchanging complaints. Patients with longstanding, stable complaints might also have a better recollection of the complaints they used to have before treatment. A comparable study of Jorritsma et al. [15] used a follow-up period of three to five months, and reported a MIC of 3.5. Although there is no information to explain the lower estimate of the MIC in our study this finding only underlines our conclusion that estimates of the MIC are not invariable and are likely to be population specific.

### Strengths and limitations

The main strength of our study lies in the use of a single study to assess the influence of different anchors, different definitions of improvement on the anchor and of population characteristics on estimates of the SDC and the MIC. Using differently phrased anchors for pain and for function gave us the opportunity to study the influence of the phrasing of the anchor in estimating the SDC and the MIC for the NDI in the same population, thereby excluding the possibility of sampling bias. A possible weakness lies in the number of recruited patients. This number of patients was enough for the main analyses, but the sample size may have been relatively small for our subgroup analyses.

Another limitation lies in the methods we use to calculate the MIC. We calculate the MIC by comparing the change score with the global perceived effect. This global perceived effect has been reported to correlate stronger with present status than with change in status [29]. A patient with severe disability needs a large improvement to arrive at a better present status after treatment and could still end up in the group of patients reporting to be unchanged even with a strong improvement of the NDI score. A patient with a low baseline score but no real change after treatment will still have a good present status at follow-up and could end up in the group of improved patients while the NDI change score is small. This could explain higher estimates of the MIC for patients with higher baseline scores and does not necessarily reflect a real need for a larger improvement of the NDI score, but could be a shortcoming of our way of calculating the MIC using a global perceived effect as the external anchor.

### Further study

It would still be interesting to study psychometric properties of the NDI in different populations, for example in patients with Whiplash Associated Disorders (WAD), and with larger sample sizes in the subgroups. It would also be very interesting to study the suggested shorter versions with an improved factor structure or to compare NDI scores with other legacy instruments or with recently developed IRT item banks for pain behaviour and pain interference [30,31].

In terms of methodology future studies may focus on alternatives for the GPE. It is quite understandable that different patients have different perceptions of what magnitude of effect they consider an important change, perhaps also depending upon the treatment administered. A treatment that is costly, painful, or strenuous might need a larger effect to be considered worthwhile, and a patient who has experienced severe side effects might even consider a large improvement not worthwhile. The development of other methods to estimate sufficient important change could lead to new perspectives. We could still make progress in defining clinical relevance [32,33].

## Conclusions

We studied the influence of the type of anchor and the definition of improvement on the estimates of the SDC and the MIC of the NDI in a sample of patients with general, non-specific, chronic neck pain. The use of two different types of anchor and two definitions of improvement only had a minimal influence on these estimates. We also estimated the SDC and the MIC in subgroups of patients with or without radiating pain and with different baseline scores. Subgroups of patients with or without radiating pain had comparable estimates of the SDC and the MIC, but subgroups of patients with different baseline scores had different estimates of the SDC and the MIC, showing that these values can be influenced by population characteristics. It is therefore concluded that we cannot readily adopt a single change score to define relevant change by combining the results of previous studies. In line with other studies we also report the SDC to be much higher than the MIC, which is a limitation of the NDI. Quite a large change score is needed to reliably label a patient as improved, raising questions about the ability of the NDI to assess change in individual patients.

## Competing interests

First author’s position at the EMGO+ Institute for Health and Care Research is funded by the Dutch Association for OrthoManual Medicine (NVOMG).

## Authors’ contributions

WS designed the study, carried out the analyses and is corresponding author, RJ carried out the study and collected the data, RWJGO and HCWdeV advised in the analyses and contributed strongly to the text. All authors read and approved the final manuscript.
